# Identification of Phenolic Compounds and Determination of Antioxidant Activity in Extracts and Infusions of Salvia Leaves

**DOI:** 10.3390/ma13245811

**Published:** 2020-12-19

**Authors:** Sławomir Francik, Renata Francik, Urszula Sadowska, Beata Bystrowska, Agnieszka Zawiślak, Adrian Knapczyk, Abdul Nzeyimana

**Affiliations:** 1 Department of Mechanical Engineering and Agrophysics, Faculty of Production Engineering and Energetics, University of Agriculture in Krakow, Balicka 120, 30-149 Krakow, Poland; slawomir.francik@urk.edu.pl (S.F.); adrian.knapczyk@urk.edu.pl (A.K.); 2 Institute of Health, State Higher Vocational School in Nowy Sacz, Staszica 1, 33-300 Nowy Sacz, Poland; 3 Department of Bioorganic Chemistry, Chair of Organic Chemistry, Faculty of Pharmacy, Jagiellonian University Medical College, Medyczna 9, 30-688 Kraków, Poland; 4 Department of Machinery Exploitation, Ergonomics and Production Processes, Faculty of Production Engineering and Energetics, University of Agriculture in Krakow, Balicka 116 B, 30-149 Krakow, Poland; urszula.sadowska@urk.edu.pl; 5 Department of Toxicology, Collegium Medicum, Jagiellonian University, Medyczna 9, 30-688 Kraków, Poland; beata.bystrowska@uj.edu.pl; 6 Department of Biotechnology and General Food Technology, Faculty of Food Technology, University of Agriculture in Krakow, Balicka 122, 30-149 Krakow, Poland; agnieszka.zawislak@urk.edu.pl; 7 Department of Biochemistry, Faculty of Science, Ege University, Izmir 35040, Turkey; nzeyimana20@gmail.com

**Keywords:** antioxidants, chromatographic analysis, DPPH, FRAP, phenols, *Salvia officinalis*

## Abstract

The influence of harvest period and drying method of *Salvia officinalis* L. leaves on the content of essential oils, polyphenols and antioxidant properties was investigated. Sage leaves were collected twice during plant blossoming (in June and July) and dried traditionally in natural conditions and at 35 °C. Antioxidant activity was assayed in methanol-acetone extracts and infusions of dried leaves with the use of free radical scavenging activity (DPPH) and ferric reducing antioxidant power (FRAP) technique. Total phenolic content in extracts as well as in infusions was determined by the means of Folin–Ciocalteu method. Based on the LC/MS analysis, the polyphenol compounds present in both extracts and in infusions were identified. The extracts contained more polyphenols and were characterized by higher antioxidant activity than infusions. In the extracts significant amount of ferulic acid was found, which was influenced both by the harvest period and drying method. The amount of ferulic acid found in extracts obtained from the June leaves dried traditionally was three times lower (6.185 μg/g DW) than in extracts from July leaves dried in the same conditions. Harvest period had a significant effect on the essential oils’ content, leaves collected in July contained 15% more oils than those collected in June.

## 1. Introduction

*Salvia officinalis* is a plant of the Lamiaceae family, widely distributed throughout the world, commonly known as sage (Dalmatian sage). It is a small, evergreen perennial plant originated from the Mediterranean region, which has been found to have not only culinary, but also health-promoting applications. Most of the papers regarding this plant are aimed at qualitative and quantitative evaluation of the components of the essential oil extracted from sage leaves [1,2,3]. In traditional medicine, Salvia is used to treat various disorders, including epilepsy, gout, inflammation, as well as diarrhea and hyperglycemia [4,5,6,7]. Scientific research suggests that *S. officinalis* has a strong antioxidant effect [8]. Enriching drinkable water with *S. officinalis* extract increases the resistance of rat hepatocytes to oxidative stress [9].

According to the Pharmacopoeia, pharmaceutical raw material of medicinal sage is the leaves and leafy tops of shoots, which should be harvested from non-flowering plants and dried. Natural drying at a temperature below 35 °C in a shaded place is preferable [10]. Hamrouni et al. [11] studied the effect of seven drying methods on the content of polyphenols in methanol extracts prepared from sage leaves.

Herbal plants such as sage have the potential to phytoremediate soils contaminated with heavy metals [12,13]. Sung and Honermeier [14] conducted a study of the effect of sage cultivation methods and harvesting timing on the yield and quality of the sage roots. They showed that there are statistically significant differences between the cultivation methods used.

The results of the work of Mameli et al. [15] also show that the biomass of crops is influenced by irrigation conditions, which result in the amount of essential oils.

In the studies by Zawiślak and Dyduch [3], conducted for sage cultivar *Bona*, the effect of the harvest period on the content of active substances in the essential oil obtained from the leaves was determined. The study also determined the quantitative and qualitative composition of essential oils obtained from the crops harvested in May and September.

Recently, more and more experiments are carried out on the use of extracts obtained from various plants for nutritional and industrial purposes. Attention is paid to aromatic plants with proven antibacterial and antioxidant properties as an alternative to environmentally safe phytoremediation of soils [16], which can be used in fields other than medicine.

The study assessed the effect of two methods of harvesting and drying essential oils from sage leaves of cv. *Bona* on the content and composition of polyphenolic compounds in extracts and infusions made from sage leaves. The antioxidant properties of both extracts and infusions of *Bona* sage leaves were analyzed. Polyphenolic compounds contained in extracts and infusions obtained from sage leaves harvested in June and July, which were dried in a traditional manner and at elevated temperature, were identified.

## 2. Materials and Methods

### 2.1. Reagents

Reagents such as acetonitrile and chloroform from we have purchased Merck (Darmstadt, Germany), while methanol, formic acid, hydrochloric acid, acetone, sodium carbonate, nitric acid from POCh (Katowice, Poland). Standards of 3,5-di-caffeoylquinic acid (3,5-diCQA), 3,5-dimethoxy-4-hydroxycinnamic acid (sinapinic acid), *p*-coumaric acid, ferulic acid, isorhamnetin, quercetin, rutin, catechin, and Folin-Ciocialteu reagent were obtained from Sigma-Aldrich (St. Louis, MO, USA). 

Reagents 2,2-Diphenyl-1-picrylhydrazyl (DPPH) and 3,4,5-trihydroxybenzoic acid (gallic acid) were purchased from Fluka (St. Louis, MO, USA).

Methanol was used to prepared the standard solutions, the comparison. Samples obtained stored at −20 °C. Further dilutions were prepared using methanol. The study used only chemicals of analytical grade.

### 2.2. Plant Material and Growth Conditions

The experiment was conducted in 2014 at the University of Agriculture in Krakow plantation located in the north-western part of Krakow. Sage (*Salvia officinalis* L.) of the *Bona* leaves were used as plant material. The study was conducted in the third year of sage vegetation. The research material came from 20 bushes that grew on the small experimental field research station Mydlniki (50.060° N, 19.959° E) at a spacing of 40 cm × 40 cm on soil covered with black polypropylene agrotextile (90 g·m^−2^).

The experiment was carried out on a soil with a granulometric composition of strong loamy sand (16–20% the floatable fraction). During the vegetation season, no hydration or any pesticides were used (the type of fertilization has already been given). No pesticides were used, only fertigation with a 0.2% solution of Kristallon, a multi-constituent fertilizer with micronutrients, in the middle of April was performed. In Kristalon fertilizer the percentage of N: P: K is the same and amounts to 18: 18: 18. This fertilizer is enriched with magnesium and sulfur with 3% MgO and 2% S. Therefore, fertilization with these macronutrients was: 0.36 g N, 0.36 g P_2_O_5_, 0.36 g K_2_O, 0.06 g MgO and 0.04 g S for each sage bush. This was an equivalent to fertilizing under each bush in the amount of 0.36 g, calculated as pure N, P_2_O_5_, K_2_O and 0.06 g MgO and 0.04 g S.

In Polish habitat salvia collection is carried out twice a year. The harvest was performed manually, using secateurs, with the beginning of plants’ flowering, about 10 cm above the soil surface. Then the plants were randomized and dried in natural conditions in a shaded room or in a laboratory dryer with forced convection at 35 °C. The volume of the dryer working chamber was 100 dm^3^. Drying time, to the desired humidity, at 35 °C lasted 4 days, while in natural conditions 2 weeks.

The meteorological data for the local station for the harvest year regarding rainfall and temperature are as follows: in June the sum of rainfall was 213.1 mm, the average temperature was 17.5 ± 1.6 °C. In the month of July, rainfall was 27.2 mm at the level of the temperature. 19.5 ± 1.5 °C.

Drying is the most common way of preserving fresh herbal raw material. The dried material with a water content of 100 mL kg^−1^ was stored in polypropylene bags with high barrier properties. Samples for analyses were taken randomly.

### 2.3. Extracts and Infusions Preparation

One gram of *Salvia officinalis* leaves were powdered in a pestle and extracted in a conic tube of 50 mL using 40 mL of 0.08 N hydrochloric acid in 80% methanol. Extraction by shaking was performed at room temperature for 2 h using an Elpan shaker, type 18, 358 S, (Lubawa, Poland). Then the samples were centrifuged at 4500 rpm for 10 min (centrifuge type MPW-350, Warsaw, Poland) and the supernatants were decanted.

The residues were re-extracted with 40 mL of 70% acetone in the same conditions and centrifuged as mentioned above. Both supernatants were combined and stored at temperature −20°C until analysis.

One gram of salvia leaves were brewed with 100 mL of deionized water at a temperature of 100 °C for 5 min. Infusions were filtered, cooled to 20 °C and made up to 100 mL with deionized water.

### 2.4. Essential Oil (Hydrodestilation)

Determination of the content of the essential oil from the dried material was carried out using a Clevenger apparatus (Slinap, Łódź, Poland). The raw material in the form of sage leaves was ground with a laboratory fine grinding mill before hydroxylation. Twenty grams of dried and then powdered leaves of sage were used for the test. The samples were weighed with an accuracy of 0.001 g.

The test material was then placed in a 1000 mL round-bottom flask (Slinap, Łódź, Polska) and 400 mL of distilled water was added. The sage containing flask was heated for 3 h, controlling the intensity of heating to the desired distillation rate [10]. The distillation rate was 2–3 mL/min. At the end of the distillation, the heating was closed and after 10 min the volume of oil was measured on a calibration tube. Three tests were carried out for each drying condition. The obtained samples of essential oils were then stored in glass vials at 4 °C for analysis.

### 2.5. Ferric Reducing Antioxidant Power (FRAP) Determination

The ability to reduce Fe^3+^ ions to Fe^2+^ ions in acidic environment (pH 3.6) was measured in the obtained extracts and infusions. For this purpose, the FRAP test was used, which was a modification of the Benzie and Strain’s [17]. Fe^2+^ ions in the presence of 2,4,6-tripirydylo-S-triazyne (TPTZ) forms Fe^2+^-TPTZ complexes of an intense blue color and maximum absorbance at 593 nm. For the assay, 1 mL of the reaction mixture was used, including 0.3 M acetate buffer (pH 3.6); TPTZ 0.01 M; iron (III) chloride 0.02 M. To such volume of the reaction mixture 50 µL of the tested extract or infusion was added. It was mixed, and incubated at 37 °C in water bath for 30 min. After this time in the samples, the absorbance at λ = 593 nm was measured using a JASCO V-530-MEDSON spectrophotometer, Tokyo, Japan. A blank test was performed similarly, but 50 µL of distilled water was added to the reaction mixture. The standard curve was linear for the Trolox concentration ranging from 0.003125 to 1 mmol. The antioxidant activity is expressed as millimoles of ferrous ions per liter (mM Fe^2+^/L). Measurements were carried out in triplicate.

### 2.6. Measurement of DPPH Radical-Scavenging Activity

The antioxidant properties of the investigated extracts and infusions were measured with the DPPH test based on the method described by Akter et al. [18]. Generally, DPPH in its stable radical form absorbs at 517 nm, but its absorption ability decreases when reduced by the antioxidant present in the sample. A 0.6 mM solution of 2,2-diphenyl-1-picrylhydrazyl (DPPH) was prepared in methanol. Samples (extracts and infusion) diluted 1: 5 were prepared before the assay. A fixed volume (25 µL) of each extract or infusion sample to be tested was added to 1 mL of DPPH. The solutions were mixed and incubated in the dark at room temperature for 30 min. After that time the absorbance of samples were measured at 517 nm (*As*) against blank sample (methanol) (*Ab*). As a control, absorbance of DPPH solution with 25 µL of distilled water was measured (*Ac*). The capability of tested compounds to scavenge the DPPH radical (antioxidant activity) was calculated using the following equation:

(1) $$DPPH\left[\%\right]=\frac{Ac-As}{Ac-Ab}\cdot 100$$

where: *Ac* was the absorbance of the control, *As* of the sample and *Ab* of the blank (methanol). Assays were carried out in triplicate.

### 2.7. Total Polyphenols Content (TPC)

Content of polyphenols was determined according to method described by Singleton and Slinkard [19] with some modifications. Samples of extracts were diluted (1:20) before analysis. Results were calculated as gallic acid equivalents (GA) and express as (mg/L). Measurements were done with the use of JASCO V-530–MEDSON, Tokyo, Japan spectrophotometer. Assays were carried out in triplicate.

### 2.8. Statistical Analysis

In order to assess the influence of controlled experimental factors on the values of dependent variables (describing the results of the experiment), it is necessary to use statistical inference methods. They make possible to generalize the research results from the random sample to the entire population. For this purpose, statistical hypotheses are tested. The null hypothesis is that the observed difference is entirely due to natural variability. The alternative hypothesis states that this is the result of an experiment [20].

Testing the differences between the results (values of dependent variables) in groups of measurements (samples) that were carried out for different values of factors is very often tested hypothesis. If more than two groups are compared, the most common method is analysis of variance (ANOVA).

In the event that the null hypothesis is rejected, it is necessary to perform post-hoc tests (multiple comparison tests). These tests allow to classify the means to groups that differ significantly. One of the most recommended is the Tukey’s HSD test [21,22,23,24].

In order to check whether the analyzed factors have an impact on the observed variables, a three-factor analysis of variance (ANOVA) was performed sequentially for each dependent (explained) variable. The dependent variables were: FRAP [mM Fe^2+^/L], DPPH [%], TPC [mg GAE/g DW]. The intergroup factors (independent variables) were: Harvest term (two levels: June, July), Drying method (two levels: naturally dried, dried at 35 °C), and Solution (two levels: extract, infusion).

Null hypotheses were tested:

1. The levels of the factors do not influence differentially the values of the dependent variables (experiment results),
2. There are no interactions between the factors (the response of the studied dependent variable to one factor is the same at all levels of the other factor).

Before proceeding with the analysis of variance, it was checked whether the assumptions regarding the following are met:

- normal distribution of the dependent variable within groups—the Shapiro-Wilk test was used
- homogeneity (homogeneity) of variance—Levene’s test was used

In this paper, the Tukey’s HSD (honestly significant difference) test for unequal sample size (used in many publications) was used as a post-hoc test [21,22,23,24].

Statistical analysis was carried out using the Statistica software (Dell Inc. (Tulsa, OK, USA, 2016). Dell Statistica (data analysis software system), version 13. software.dell.com). Analytical results were expressed as the means ± standard deviations (SD). *p* values < 0.05 were regarded as significant.

### 2.9. LC-MS/MS Conditions

LC was performed using an Agilent 1100 (Agilent Technologies, Waldbronn, Germany) HPLC system consisted of the following components: a pump in a gradient mode, degasser, an auto sampler with a 100 µL injection loop and DAD detector. MS/MS analysis were carried out on an Applied Biosystems/MDS Sciex (Concord, ON, Canada) API 2000 triple quadrupole mass spectrometer with an electrospray ionization (ESI) interface.

The chromatographic separation was performed in the gradient mode with a Thermo Scientific BDS HYPERSIL C18 column (100 nm × 3 mm I.D., 3 µm particle size) (Thermo Scientific, Waltham, MA, USA). The advance column, with a precolumn (10 mm × 3 mm I.D., 3 µm particle size), was set at 25 °C and a mobile phase flow rate was 0.5 mL/min. The gradient elution mobile phases consisted of 0.02 M formic acid in water (phase A) and 0.02 M formic acid in acetonitrile (phase B). The gradient began initially at 2% A in 5 min, increasing linearly to 6%, for next 22 min to 60%, then decreased to 40% at 5 min and next 5 min increased to 100% and then decreased to 2% at 2 min. Finally, the last 8 min of analysis was kept at 2% to stabilize the baseline.

The sample temperature was maintained at 4 °C in the autosampler prior to analysis. Quantification of the analysis was performed using the tandem electrospray MS in a negative mode (ESI^−^). To find the optimal parameters of the ion path and ion source of the studied compound, the quantitative optimization was done by direct infusion of the standards (at concentration of 1 µg/mL and the flow rate of 10 µL/min) using a Hamilton syringe pump (Hamilton, Reno, NV, USA). MS/MS analysis allowed recording of production spectra, monitoring each precursor [M-H^+^]^−^ion.

The dwell time that the mass spectrometer operated with was 200 ms. For each compound, the dominant production’s multiple reaction monitoring (MRM) was performed at optimal conditions. Ion source parameters were: nebulizer gas: 30 psi; ion spray voltage (IS): −4200 V; turbo gas: 10 psi; curtain gas: 20 psi; temperature of the heated nebulizer: 350 °C. Following the optimization of individual compounds, the parameters of ion path were obtained, which are presented in Table 1. The following are included among the parameters: focusing potential (FP): 200 V; collision cell entrance potential (CEP): 0 V; decluttering potential (DP): 20 V; entrance potential (EP): 10 V; collision cell exit potential (CXP): 2 V [25,26,27,28]. To confirm the identification of studied compounds the comparison of precursor/production pair *m*/*z* values and LC retention times with standards was performed on investigated samples. This comparison is presented in Table 1. The Applied Biosystems Analyst software, version 1.4.2 (Foster City, CA, USA) helped to accomplish data processing and acquisition.

## 3. Results

### 3.1. Results for Essential Oils

The contents of essential oils in leaves of *Salvia officinalis* L. of cv. *Bona* variety collected in June and July are presented in Table 2. Obtained values were influenced both by the harvest period and by the drying method of sage leaves. On average, statistically significant higher content of these essential oils were determined in leaves harvested in July dried at 35 °C (1.78 ± 0.05 mL/100 g) and in those dried under natural conditions (1.95 ± 0.01 mL/100 g) vs. leaves collected in June (dried at 35 °C 1.58 ± 0.06 mL/100 g, naturally dried 1.68 ± 0.06 mL/100 g) (Table 2).

Regarding the material harvest in July, it was found that the drying method also influenced the content of essential oils. Statistically, more oils were obtained with natural drying than with drying at 35 °C.

### 3.2. Antioxidant Parameter Results (FRAP, DPPH, TPC)

The results of the analysis of variance are presented in Table 3.

The ANOVA results (Table 3) show a statistically significant effect of the independent variable Solution on all three dependent variables, while the independent variables Harvest Term and Drying had a statistically significant effect on the dependent variables FRAP and TPC. For the FRAP and TPC variables, there is a statistically significant interaction of the Solution*Harvest Term factors.

Results of the antioxidant properties evaluation as well as total polyphenols content are presented in Table 4. FRAP values for methanol-acetone extracts obtained from sage leaves harvested in July dried naturally and at 35 °C were statistically significantly higher than for extracts from June harvest (naturally: July 496 ± 21 mM Fe^2+^/L vs. June 381 ± 64 mM Fe^2+^/L, and dried at 35 °C July 436 ± 10 mM Fe^2+^/L vs. June 300 ± 26 mM Fe^2+^/L). Moreover, the FRAP values in the case of extracts obtained from naturally dried leaves from June and July were higher than in the case of extracts obtained from leaves dried at 35 °C

Antioxidant activity of the infusions expressed as FRAP values was statistically significantly lower (276 ± 45 mM Fe^2+^/L in June, 265 ± 17 mM Fe^2+^/L in July, drying natural) than the antioxidant activity of extracts determined by the same method (381 ± 64 mM Fe^2+^/L in June, 496 ± 21 mM Fe^2+^/L in July, drying natural). There were no significant changes observed in the FRAP values for the infusions obtained. The value of the measured parameter in the case of infusions was not affected by the method of drying. The samples collected in June and July had comparable value of the measured parameter FRAP, regardless of the drying method. (Table 4).

In the case of the assessment of the ability to reduce the pool of free radicals expressed as DPPH, it was found that in extracts obtained from leaves harvested in June and July the DPPH values were significantly higher than in the infusion obtained from leaves harvested in June and dried at 35 °C. (56.2 ± 10.0% vs. 24.3 ± 5.2%). This statistically significant increase in DPPH value between the extracts and the infusion obtained from leaves harvested in June and dried at 35 °C was observed in the extracts regardless of the method of drying the plant material.

Statistically significant differences in the total polyphenol content (TPC) were observed in the extracts obtained from leaves harvested in June and July and dried naturally. In the June harvest, TPC was recorded at the level (11.6 ± 5.6 mg GAE/g DW) which was lower than the TPC value determined in the July harvest (14.9 ± 4.1 mg GAE/g DW).

In extracts obtained from leaves dried at 35 °C a statistically significant increase in the TPC parameter was observed in the July harvest (17.1 ± 7.1 mg GAE/g DW vs. 12.4 ± 6.1 mg GAE/g DW) as well. Based on the obtained results, it can be assumed that the July harvest, regardless of the drying method, contains statistically significantly more polyphenolic compounds.

### 3.3. The Results of the Chromatographic Analysis

**Figure 1:**
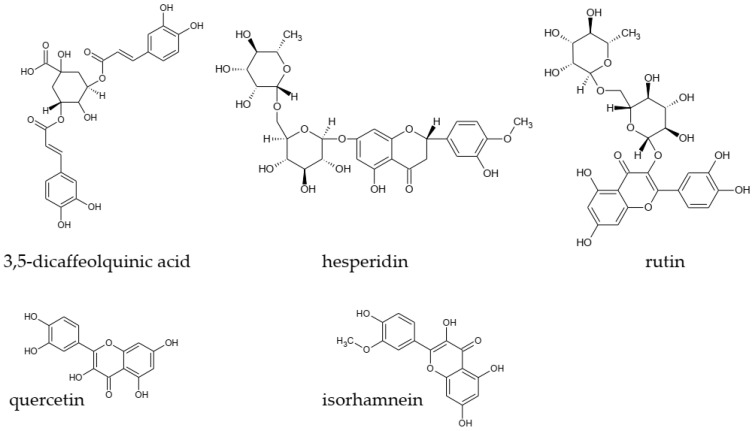
Structures of reference substances used for identification.

**Figure 2:**
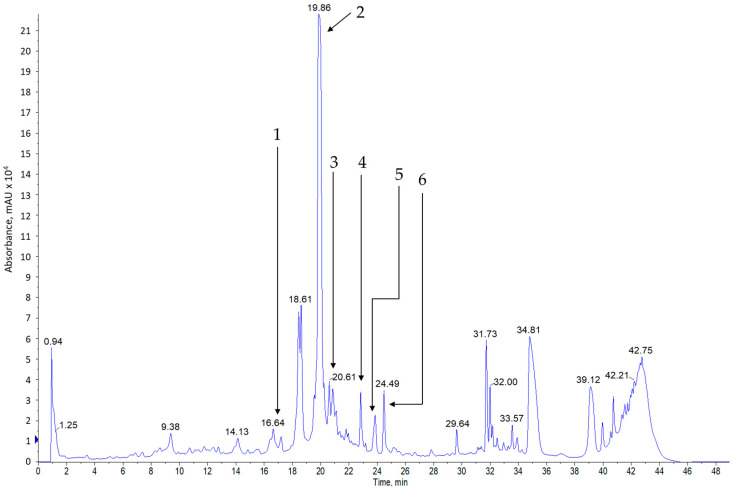
Example of DAD chromatogram for extract of salvia leaves in June dried naturally of the reference substances dissolved in methanol; (1) catechin ^13^C; (2) *p*-coumaric acid; (3) ferulic acid; (4) sinapinic acid; (5) hesperidin; (6) isorhamnetin.

**Figure 3:**
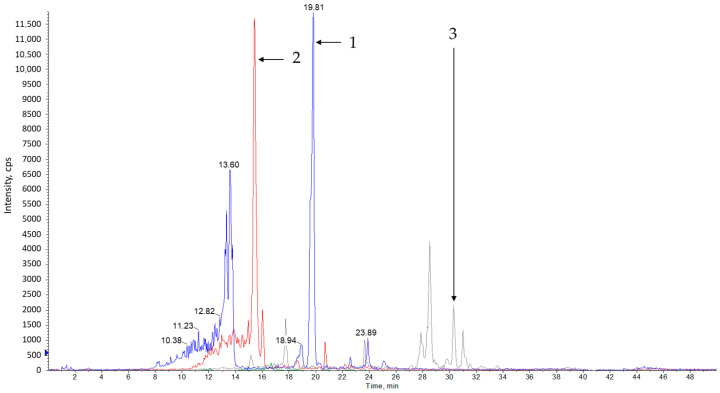
Example of a TIC chromatogram for extract of salvia leaves dried naturally. The chromatogram was obtained for the maximum absorbing wavelength at each time point; (1) *p*-coumaric acid (*m*/*z* = 162.8/119.1) and its derivatives; (2) ferulic acid (*m*/*z* = 192.8/134.0); (3) catechin (*m*/*z* = 289.0/289.0) and its derivatives.

**Figure 4:**
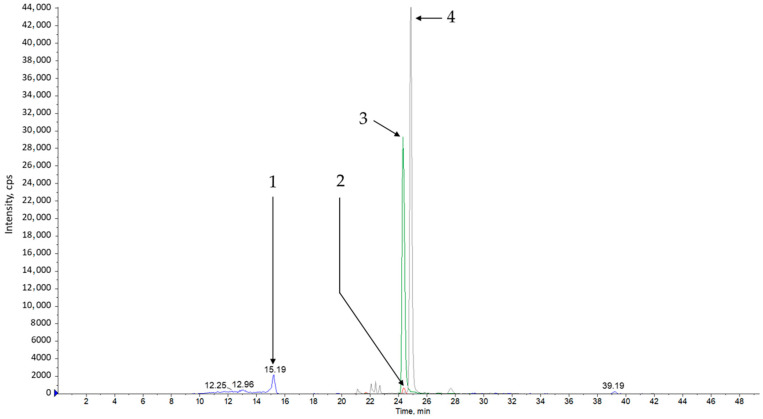
Example of chromatogram XIC obtained for a sample of extract of salvia harvest 1 drying naturally. The chromatogram was obtained for the maximum absorbing wavelength at each time point. (1) catechin C^13^ (IS) (*m*/*z* = 291.4/291.4); (2) hesperidin (*m*/*z* = 300.7/163.8); (3) quercethin (*m*/*z* = 301.03/178.9); (4) isorhamnetin (*m*/*z* = 314/300).

Analysis of methanol extracts from sage leaves harvested in June and July and dried naturally or at 35 °C showed that the tested material contains many polyphenol compounds. The compounds presented in Table 5 and Figure 1, Figure 2, Figure 3 and Figure 4 were identified in all of the tested samples.

It was found that a significant factor influencing the content of 3,5-dicaffeoylquinic acid (3,5-dCQA) in the extracts and infusions was the harvest time. Higher concentration of this compound was determined in the material coming from second harvest. A significant amount of this acid was assayed in extracts made from leaves dried at 35 °C in comparison to extracts prepared from sage leaves dried in natural conditions.

No such differences were observed in the infusions in the case of 3,5- dicaffeoylquinic acid content depending on the drying temperature. However, there was a significant difference in the 3,5-dCQA content depending on the harvest time. The leaf infusions harvested in July contained more of this acid than the leaf infusions harvested in June.

A similar relationship between the amount of extracted compound and drying conditions was observed in the case of majority of assayed polyphenols (Table 5).

Leaf extracts collected in July contained twice the amount of sinapinic acid in comparison to extracts obtained from leaves collected in June. In the case of infusions it was observed that the content of this acid is higher in the material collected in July as well. Similar relationships were observed for *p*-coumaric acid. Its content was influenced by the harvest time. In extracts made from leaves harvested in July, more of this compound was determined than in extracts made from leaves harvested in June.

The content of ferulic acid in the extracts was influenced by the time of leaf harvesting and drying methods. Increased amounts were observed in samples of extracts obtained from leaves harvested in July and naturally dried. The harvest time influenced the content of this acid in the infusions, but the drying method was not significant. In the case of hesperidin, its content was higher in the extracts obtained from the July harvest, but the content of this compound in the infusions depended on the drying method, and was significantly higher in the case of drying leaves at 35 °C.

In the case of the content of such compounds as isorhamnetin, catechin, rutin, quercetin in the extracts, increased amounts were observed for the June harvest. The method of drying influenced the content of isorhamnetin and rutin. In the case of isorhamnetin, the content of this polyphenol was higher in the samples of extracts obtained from leaves dried at 35 °C, in the case of rutins, the opposite was true. The greater amount of rutin was found in the samples obtained from the leaves after natural drying.

## 4. Discussion

Medicinal sage (*S. officinalis*) is one of the 900 species that have been found to be widely used in herbal medicine [29]. One of the reasons why other sage species have not yet been recognized as medicinal plants is accurate diagnosis and selection of the mode of their active functions. Systematic chemical analysis of different species of sage, including new variety *Bona*, and comparing it with the composition of medical sage can help find new further treatments.

A great number of plant extracts are characterized by antioxidant properties. *Salvia officinalis* L. is known mainly as a plant with strong antiseptic, anti-inflammatory and astringent features. Antioxidant activity plays an important role in fighting against certain diseases and many researches have demonstrated the antioxidant role of phenolic acids [30].

The chemical diversity of antioxidants contributes to difficulties in separating and quantifying these components in plant material. It is, therefore, desirable to establish a method that can be used to measure the total antioxidant activity directly in plant extracts [31]. In the past two decades there has been observed an increasing interest in researches concerning prevention of uncontrolled oxidation leading to various diseases in a living organism [32]. Studies have shown the crucial role of oxidative stress in the causes and progression of various diseases, including AIDS, aging, arthritis, asthma, autoimmune diseases, carcinogenicity, cardiovascular disorders, cataracts, diabetes, neurodegenerative diseases, Alzheimer’s and Parkinson’s diseases [33,34,35,36,37,38]. Clinical studies have confirmed that *S. officinalis* improves cognitive performance in both healthy participants and patients with cognitive impairment or dementia [39].

Moss et al. demonstrated that Salvia essential oil is likely to affect the level of memory in healthy adults [40,41]. Scholey et al. observed that the ethanol leaf extract of *S. officinalis* improved memory of healthy elderly people [42]. Therefore, there has been a continuous search for antioxidants naturally found in plants as alternatives to synthetic compounds.

Phenolic acids are one of the most numerous groups of compounds with antioxidant activities. These compounds take part in defensive processes during infection, excessive sun exposure and injuries in plants [43]. Based on the results of this study, it can be stated that the leaves of the *Bona* sage can be considered as a rich source of polyphenolic compounds such as phenol acids, flavanols and flavonoids.

Antioxidant effect of phenolic acids is closely related to their chemical structure. The amount of hydroxyl groups and their position in the aromatic ring determines their activity. The strongest of these, are acids which substituents are in ortho- or para- position. Introduction of the group of electrons’ donor in the ortho- position, alkyl or methoxyl, increases the stability of the antioxidant properties of phenolic acids [44]. Sinapinic acid with two methoxyl groups is more active than ferulic acid with one methoxyl group and the last one is more active than cumaric acid (containing one hydroxyl group, Figure 1). The study showed that significant amounts of sinapinic acid were present in the sage infusions. The content of this acid depended on the harvest date and drying method. A much greater amount of this compound was found in infusions and extracts obtained from sage leaves harvested in July and dried at 35 °C. Although ferulic acid is less active, its concentration is significantly higher compared to the content of sinapinic or *p*-coumaric acid (Table 5).

Studies conducted on ferulic acid have shown that this compound has a very strong antioxidant activity, as well as it shows capability to enhance activity of enzymes that neutralize free radicals, and inhibiting the activities of enzymes involved in their catalysis [45,46,47]. Due to the high ferulic acid content in the extracts obtained from second harvest, it can be assumed that this compound could promote the increase in FRAP and DPPH values. Caffeic acid derivatives such as 3,5-dCQA have strong antiviral properties [47]. In this work, there was observed that 3,5-dCQA was present in greater amounts in infusions than in extracts. The harvest time also had an effect on its content. Engelsma [48] showed that in the light period the content of phenol (mainly chlorogenic and isochlorogenic acid) increases proportionally to the length of this period, which is also confirmed by our research.

In the tested samples it was also discovered that the extracts and infusions contained flavanols (catechin) and flavonols (rutin, quercetin). Bioavailability of flavonols also depends on their chemical structure. Quercetin and its glycosides have the highest absorption rate among the polyphenolic compounds tested [49]. In the studied material, sage leaves of cv. *Bona*, the quercetin content was higher in extracts from sage leaves than in infusions. It was observed that higher amounts of this compound were found in leaves harvested in June.

Flavonoids are a group of substances which biological activity is primarily associated with blood vessels and the circulatory system. These compounds exhibit excellent properties of blood vessels protection, thus acting anti-inflammatory. Such a substance is, among others, rutin, which was found both in extracts and infusions made from sage leaves of the *Bona* variety.

The rutin content depended on the method of drying the leaves. In the case of rutine, natural drying promoted the growth of this compound in both extracts and infusions. The leaves harvested in June have also been found to contain higher amounts of these flavonoids. It has been observed that a greater amount of rutin was found in infusions than in extracts.

The polyphenol composition for some *Salvia* L. species was also analyzed by Hanganu et al. [50]. They determined the composition of the ethanolic extract obtained from aerial parts of plants. As in the *Bona* variety analyzed in our study, they found that the polyphenol profile for Salvia L contains common components. These are caffeic acid, *p*-cumaric acid, as well as other rare compounds such as ferulic acid, rutin, and quercetin. All studied species of sage contain significant amounts of polyphenolic compounds and may constitute an important source of natural antioxidants.

When preparing extracts and infusions, the harvest period and drying methods of leaves should be considered. Drying in natural conditions resulted in better antioxidant properties of sage leaves compared to material dried at 35 °C (higher values of FRAP and DPPH), whereas total polyphenol content was greater in samples dried at higher temperature. Obtained values may be the result of transformations in phenolic compounds that may occur under elevated temperatures or formation of new compounds that react with Folin’s reagent. The effect of harvest time on these properties was also observed. The harvest carried out in July resulted in an increase in the antioxidant capacity of the test material and also had a positive effect on the polyphenol content.

Santos-Gomes and Fernandes-Ferreira [51] draw attention to the seasonal variability of essential oils content in sage. The presented data on the higher content of essential oils obtained from the harvest in July are consistent with the results of Baydar et al. [52], who similarly received the maximum content of essential oils in medical sage in July. In the cited studies, the analyzes were carried out over four months from June to September. Bettaieb et al. [53] found that moderate water scarcity may be a factor increasing the content of essential oils in medical sage. The average rainfall in the analyzed period in July was 27.2 mm, while in June it was 213.1 mm. Moreover, the average daily temperatures in the studied months differed. July was characterized by a higher average temperature (19.4 °C) compared to June (17.5 °C), which may also be a factor increasing the content of essential oils.

As pointed out by Farhat et al. (2009) [54] *Salvia officinalis* can be a valuable natural source of antioxidants that can be used both in medicine and in the food industry, so you should try to optimize their content.

Most of the identified polyphenols were present in both extracts and infusions obtained when drying the leaves at 35 °C (3.5-dCQA, sinapinic acid, *p*-coumaric acid, isorhamnetin and catechin). The remaining polyphenols, i.e., ferulic acid, hesperidin and rutin, were found in greater amounts in the extracts obtained from naturally dried leaves. This observation may be important when planning the extraction of these compounds from plant matter.

## 5. Conclusions

The results obtained in this study have proved that *Salvia officinalis* variety *Bona* is a promising source of antioxidants and can be used as a prevention against illnesses caused by oxidative stress. In addition, plant extracts can be used as readily available source of natural antioxidants and possibly food supplements or in the pharmaceutical industry. When preparing extracts and infusions, the harvest time and methods of drying plant material should be considered.

On the basis of the obtained results, it can be assumed that the July harvest, regardless of the drying method, contains significantly more polyphenolic compounds.

Consequently, sage leaves of the *Bona* variety can be used as food additives that are a source of phytochemicals. There is a need for further research on this variety and its possible use in the production of new functional foods.

## Figures and Tables

**Table 1:** The ion path parameters obtained following individual compounds optimization.

Compound	M * [g/mol]	*m*/*z* [M-H^+^]^−^	*m*/*z* MRM *	RT [min]	DP **	FP ***	CE ^#^	CXP ^##^
3,5-dCQA ^1^	516.45	515.40	352.80	22.36	−70	−100	−25	−10
sinapinic acid	223.21	222.10	207.80	20.88	−31	−160	−12	−15
*p*-coumaric acid	163.16	162.80	119.10	19.81	−31	−320	−9	−22
ferulic acid	193.18	192.80	134.00	20.70	−36	−250	−10	−8
hesperidin	609.56	608.14	163.80	24.36	−86	−50	−15	−6
isorhamnetin	315.26	314.70	300.00	24.80	−91	−340	−16	−10
catechin	290.27	289.27	a ^2^	15.15	−20	−200	−15	−15
rutin	610.52	609.14	301.00	20.64	−20	−200	−29	−15
quercetin	302.24	301.03	178.90	24.30	−20	−200	−15	−15

* M-Molecular mass; [M-H^+^]^−^ ion; MRM multiple reaction monitoring; RT-Retention time; ** DP decluttering potential; *** FP focusing potential; ^#^ CE collision energy; ^##^ CXP collision cell exit potential; ^1^ 3,5-dCQA–3,5-diCaffeoylquinic acid; ^2^ a-fragmentation has not been made.

**Table 2:** Effect of harvest period and drying method on essential oils content of sage leaves.

Treatment	Essential Oils [mL/100 g]
Harvest June, dried naturally	1.68 ± 0.06 ^BC^
Harvest June dried at 35 °C	1.58 ± 0.06 ^C^
Harvest July, dried naturally	1.95 ± 0.01 ^A^
Harvest July, dried at 35 °C	1.78 ± 0.05 ^B^

The number of independent tests done in Clevenger device for each sample was three. Data are presented as mean values of three independent measurements ± standard deviation (SD). ^A, B, C, BC^ Different letters in the same columns indicate significant differences according to Tukey’s test (*p* < 0.05).

**Table 3:** ANOVA results for FRAP, DPPH and TPC parameters in samples of sage leaves collected in June and July.

Solution	*p* FRAP [mM Fe^2+^/L]		*p* DPPH [%]		*p* TPC [mg GAE/g DW]	
Solution	0.00000	*	0.00001	*	0.00000	*
Harvest Term	0.00015	*	0.06366		0.00002	*
Drying	0.00118	*	0.08957		0.00516	*
Solution * Harvest Term	0.00106	*	0.11727		0.01057	*
Solution * Drying	0.30904		0.70735		0.88394	
Harvest Term * Drying	0.22673		0.11428		0.94231	
Solution * Harvest Term * Drying	0.62503		0.96881		0.22557	

Significant results are indicated by asterisks * (*p* < 0.05).

**Table 4:** Effect of harvest period and drying method on antioxidant activity indicators (FRAP, DPPH and TPC) marked in salvia leaves.

Solution	Harvest Term	Drying Method	FRAP [mM Fe^2+^/L]	DPPH [%]	TPC [mg GAE/g DW]
extract	June	natural	381 ± 64 ^B,C^	57.5 ± 4.4 ^B^	11.6 ± 5.6 ^A,B^
extract	June	35 °C	300 ± 26 ^A,B^	50.9 ± 9.4 ^A,B^	12.4 ± 6.1 ^B,C^
extract	July	natural	496 ± 21 ^D^	60.5 ± 8.8 ^A,B^	14.9 ± 4.1 ^C,D^
extract	July	35 °C	436 ± 10 ^C,D^	56.2 ± 10.0 ^A,B^	17.1 ± 7.1 ^D^
infusion	June	natural	276 ± 45 ^A^	36.0 ± 7.0 ^A,C^	8.9 ± 6.8 ^A^
infusion	June	35 °C	211 ± 38 ^A^	24.3 ± 5.2 ^C^	10.2 ± 5.9 ^A,B^
infusion	July	natural	265 ± 17 ^A^	42.2 ± 4.9 ^A,B,C^	10.6 ± 4.9 ^A,B^
infusion	July	35 °C	249 ± 22 ^A^	40.4 ± 8.1 ^A,B,C^	11.6 ± 1.9 ^A,B^

^1^ Data are presented as means from independent measurements ± standard deviation (SD). Different letters in the same columns indicate significant differences according to Tukey’s test (*p* < 0.05).

**Table 5:** Effect of harvest period and drying method on content of phenolic compounds in salvia leaves.

	Solution-Harvest Term-Drying Method							
Compound	Extract -June- Natural	Extract -June- 35 °C	Extract -July- Natural	Extract -July- 35 °C	Infusion -June- Natural	Infusion -June- 35 °C	Infusion -July- Natural	Infusion -July- 35 °C
3,5-dCQA ^1^ [ng/g DW]	9.198	29.286	18.333	40.834	33.659	35.06	47.944	48.643
sinapinic acid [μg/g DW]	0.199	0.358	0.539	0.621	0.514	0.711	0.588	0.782
*p*-coumaric acid [μg/g DW]	0.21	0.258	0.564	0.602	0.517	0.711	0.486	0.442
Ferulic acid [μg/g DW]	6.185	2.897	19.396	9.236	1.884	2.59	4.177	5.588
hesperidin [ng/g DW]	1.667	1.049	3.866	3.53	3.601	6.459	1.072	5.352
isorhamnetin [ng/g DW]	3.617	5.558	2.333	3.161	0.595	0.731	0.451	0.521
catechin [ng/g DW]	0.384	0.419	0.265	0.314	0.537	0.439	0.344	0.297
rutin [μg/g DW]	11.49	6.712	9.13	6.653	13.293	7.758	10.131	5.925
quercetin [μg/g DW]	0.272	0.21	0.184	0.228	0.269	0.193	0.061	0.034

^1^ 3,5-dCQA–3,5-diCaffeoylquinic acid; DW—dry weight.
